# Macrolide Therapy in Adults and Children with Non-Cystic Fibrosis Bronchiectasis: A Systematic Review and Meta-Analysis

**DOI:** 10.1371/journal.pone.0090047

**Published:** 2014-03-06

**Authors:** Yong-hua Gao, Wei-jie Guan, Gang Xu, Yan Tang, Yang Gao, Zhi-ya Lin, Zhi-min Lin, Nan-shan Zhong, Rong-chang Chen

**Affiliations:** State Key Laboratory of Respiratory Diseases, National Clinical Research Center for Respiratory Disease, Guangzhou Institute of Respiratory Diseases, The First Affiliated Hospital of Guangzhou Medical University, Guangzhou, Guangdong, China; Queens University Belfast, Ireland

## Abstract

**Background:**

A systematic review and meta-analysis was conducted to evaluate the efficacy and safety of macrolide therapy in adults and children with bronchiectasis.

**Methods:**

We searched the PUBMED, EMBASE, CENTRAL databases to identify relevant studies. Two reviewers evaluated the studies and extracted data independently. The primary outcome was the number of bronchiectasis exacerbations. Secondary outcomes included exacerbation-related admissions, quality of life (QoL), spirometry, 6-minute walk test (6MWT) and adverse events.

**Results:**

Nine eligible trials with 559 participants were included. Six were conducted on adults, and the remaining on children. Macrolide therapy significantly reduced the number of patients experiencing one or more exacerbation in adults [risk ratio (RR) = 0.59; 95% CI, 0.40 to 0.86; P = 0.006; I^2^ = 65%] and children [RR = 0.86; 95% CI, 0.75–0.99; P = 0.04; I^2^ = 0%], but not the number of patients with admissions for exacerbation. Macrolide therapy was also associated with reduced frequency of exacerbations in adults (RR = 0.42; 95% CI, 0.29 to 0.61; P<0.001; I^2^ = 64%) and children (RR = 0.50; 95% CI, 0.35 to 0.71; P<0.001). Pooled analyses suggested that spirometry, including FEV_1_ and FVC, were significantly improved in adults but not in children. Macrolide therapy improved the QoL (WMD, −6.56; 95% CI, −11.99 to −1.12; P = 0.02; I^2^ = 86%) but no significant difference in 6MWT (WMD, 4.15; 95% CI, −11.83 to 20.13; P = 0.61; I^2^ = 31%) and the overall adverse events (RR, 0.96; 95% CI, 0.82 to 1.13; P = 0.66; I^2^ = 0%) in adults. However, reports of diarrhea and abdominal discomforts were higher with macrolide therapy.

**Conclusions:**

Macrolide maintenance therapy, both in adults and children, was effective and safe in reducing bronchiectasis exacerbations, but not the admissions for exacerbations. In addition, macrolide administration in adults was associated with improvement in QoL and spirometry, but not 6WMT. Future studies are warranted to verify the optimal populations and clarify its potential effects on antimicrobial resistance.

## Introduction

Bronchiectasis is a chronic and etiologically heterogeneous airway inflammatory disease characterized by productive cough, recurrent infective exacerbations and impaired quality of life (QoL) [1]. The diagnosis and treatment of bronchiectasis has rarely been focused on, presumably due to the misperception that it is rare and can be readily managed once identified [1], [2]. Although the true prevalence and burden are still unknown, recent epidemiological surveys demonstrated that the prevalence and the resultant hospitalizations are steadily increasing worldwide [3]–[7]. Unfortunately, current managements are mostly based on expert consensus or extrapolation from other respiratory diseases, i.e. cystic fibrosis (CF) and chronic obstructive pulmonary disease (COPD) due to a paucity of evidence-based data [1]. To date, effective long-term treatments, apart from chest clearance techniques, are lacking [1]. The development of evidence-based treatment is urgently needed to reduce the growing clinical burden of this ‘orphan’ disease [1], [2].

Macrolides, which have considerable anti-inflammatory and immunomodulatory properties in addition to their antibacterial effects, might be effective in CF, COPD, asthma, diffuse panbronchiolitis and post-transplant bronchiolitis obliterans [8]–[13]. The precise mechanisms of action are unclear, but it might be associated with attenuated production of pro-inflammatory cytokines, airway mucus secretion and viscosity [14]. This provided the rationale for use in patients with non-CF bronchiectasis. Several trials have been performed to evaluate the effectiveness of macrolides for bronchiectasis maintenance treatment [15]–[17]. However, individual studies on the benefits of prevention from exacerbations, other clinical crucial end-points and safety of macrolides in bronchiectasis patients were of limited sample size and marked heterogeneity of results.

We conducted a systematic review and meta-analysis to evaluate the impacts of macrolides on the number of bronchiectasis exacerbations and other clinical measures, i.e. admission for exacerbations, QoL, spirometric indices and adverse events.

## Materials and Methods

### Literature search

We performed a literature search using PUBMED, EMBASE and CENTRAL databases for relevant studies published up to December 5^th^, 2013. An English language restriction was imposed. Search terms included “Macrolides”, “Macrolide*”, “Azithromycin”, “Erythromycin”, “Clarithromycin”, “Roxithromycin”, “Troleandomycin”, “Bronchiectasis”, “Bronchiect*”. Searches were limited to human only and randomized controlled trials (RCTs). Additionally, we screened the reference lists of the papers identified through database search for other potentially eligible studies.

### Study eligibility

Two reviewers independently (Y. G. and W.G.) performed the study selection, with differences resolved by mutual discussion and arbitration of a third reviewer (G.X.), if necessary. Studies that met the following criteria were considered potentially eligible: (1) Study design: RCTs; (2) Populations: clinically stable non-CF bronchiectasis defined by high-resolution computed tomography (HRCT); (3) Intervention: long-term macrolide treatment (≥2 months); (4) Comparison on interventions: placebo or usual care; (5) Outcomes: the primary outcome was the number of bronchiectasis exacerbations including the total number of patients experiencing one or more exacerbations as well as the frequency of exacerbation in the study period, and secondary outcomes included admissions for infective exacerbations, QoL, spirometric indices, sputum volume, 6-minute walk test (6MWT) and adverse events.

For studies reported in two or more publications, only the most complete publication was used for data extraction. Abstracts published in scientific conferences or website materials were excluded, for these studies had not been peer-reviewed and their inclusion might have biased the meta-analysis.

### Data extraction

Two authors independently reviewed eligible studies and extracted the following data: first author; year of publication; study design; number of participants per treatment arm; type of macrolides, dose and duration; inclusion/exclusion criteria; length of follow-up and clinical outcomes. Any differences on data extraction were resolved by discussion and consultation of the third author (G.X.), if appropriate. Data were mainly obtained from original manuscripts when possible; when data were insufficient, we contacted the authors by e-mail, or obtained estimates from the previous meta-analyses on the topic if available [18].

### Quality assessment

Two reviewers (Y. G. and W.G.) independently assessed the methodological quality of RCTs by using a validated scale (Jadad scoring system) [19] to determine how the randomization sequence was generated, how allocation was concealed and how missing outcome data were reported and analyzed, thus giving rise to a score of 0–5. Studies with a score of 3 or more were of high quality and otherwise low quality. Although concealed treatment allocation was not part of this rating scale, it was included in quality assessment. Any disagreements regarding the risk of bias assessments were harmonized by discussion and consensus.

### Statistical analysis

An intervention meta-analysis was conducted using Review Manager Software 5.1.2 (Cochrane Collaboration, Oxford, UK) and STATA 12.0 software (Stata Corporation, College Station, TX, USA). Risk ratios (RR) for dichotomous variables and weighted mean difference (WMD) or standard mean differences (SMD) for continuous variables with 95% confidence intervals (95% CI) were calculated; SMD were used when studies reported different units or scales for the outcome. We measured heterogeneity by using the I^2^ test [20], with suggested thresholds for low (25%-50%), moderate (50%–70%) and high (>75%) heterogeneity, respectively. Study-level data were pooled using random-effect models because of the anticipated heterogeneity with different macrolides used, different durations of therapy, different study designs and populations. For the potential sources of heterogeneity, sensitivity analyses were conducted to explore the influence of alternative statistical models (fix-effects model and random-effects model) and excluding studies with low quality on the findings. Publication bias was assessed by a funnel plot using exacerbations as an endpoint. The Egger's test was used to evaluate publication bias statistically. P<0.05 was considered statistically significant.

For primary outcome, subgroup analyses were also performed based on: (1) duration of treatment: ≥6 months vs. <6 months; (2) type of macrolides: azithromycin vs. erythromycin; (3) type of control group: placebo vs. usual care; (4) location of the country.

## Results

### Literature review

**Figure 1:**
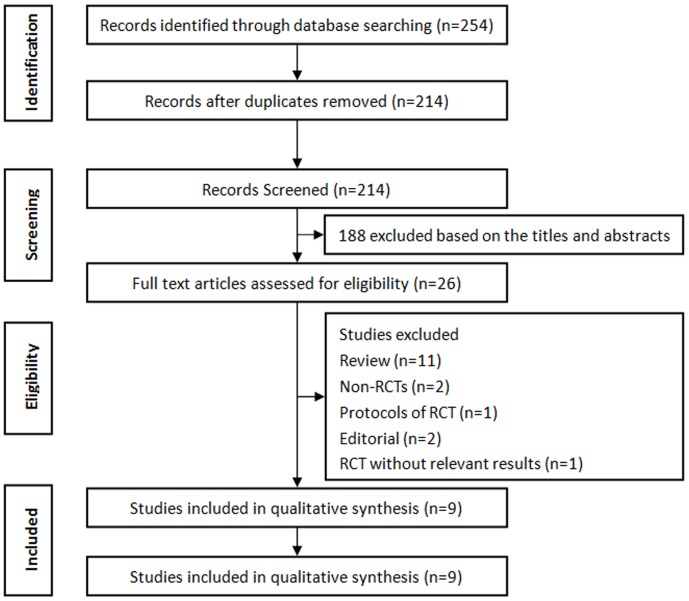
Flow of study selection.

Initial literature searches retrieved 254 articles, from which 214 were screened after excluding duplicates. Following screening of the titles and abstracts, 188 citations were removed due to irrelevant publishing types or studies. Of 26 full-text citations, 9 with 559 participants fulfilled inclusion criteria [15]–[17], [21]–[26]. Figure 1 describes the flow chart of our systematic review. The included studies were published between 1997 and 2013. Eight of the trials were parallel group studies [15]–[17],[21],[22],[24]–[26] and one cross-over study [23]. Six of these studies recruited patients with adults [15]–[17], [22], [23], [25] and three with children [21], [24], [26]. Five trials evaluated azithromycin [16], [17], [23], [25], [26], three erythromycin [15], [22], [24] and one roxithromycin [21]. Seven trials were placebo-controlled trials [15]–[17], [21], [22], [24], [26], whereas the control groups comprised usual medication care in the remaining trials [23], [25]. The duration of treatment ranged from 8 weeks to 24 months. All trials reported exacerbations as either dichotomous or continuous outcomes, with six using exacerbations as primary outcomes [15]–[17], [23], [24], [26]. The characteristics of the included trials are shown in Table 1.

**Table 1:** Characteristics of randomized clinical trials included in the meta-analysis.

Study/Year	Study Design	Number of subjects	Macrolides/dose/duration	Inclusion criteria/exclusion criteria	Length of follow-up
Koh YY [21]/1997	DB, RCT	25(13 treatment, 12 placebo)	Roxithromycin/(4 mg/kg) twice daily/12 weeks	**Inclusion:** Children, stable bronchiectasis, increased AR **Exclusion:** bronchopulmonary aspergillosis, CF, humoral immune deficiency, using sodium cromolyn or ICS, URTI or using antibiotics or corticosteroid in the past 1 month	12 weeks
Tsang KW [22]/1999	DB, RCT	21(11 treatment, 10 placebo)	Erythromycin/500 mg twice daily/8 weeks	**Inclusion:** 24-hr sputum>10 ml, stable idiopathic bronchiectasis. **Exclusion:** unreliable clinical attendance, adverse reactions to macrolides, lactating females.	8 weeks
Cymbala AA [23]2005	Open label, crossover, RCT	22(11 subjects, crossover)	Azithromycin/500 mg twice weekly/6 months	**Inclusion:** Age≥18 years. **Exclusion:** intolerance or allergy or sensitivity to macrolides, unable to follow instructions.	6 months
Wong C [17]2012	DB, RCT	141(71 reatment, 70 placebo)	Azithromycin/500 mg three times weekly/6 months	**Inclusion:** Age>18 years, stable bronchiectasis, 1 or more exacerbation requiring antibiotic therapy in the past year. **Exclusion:** CF, hypogammaglobulinaemia, ABPA, positive culture of NTM in the past 2 years or at screening, macrolide treatment for more than 3 months in the past 6 months, unstable arrhythmia	12 months
Altenburg J [16]/2013	DB, RCT	83(43 treatment, 40 placebo)	Azithromycin/250 mg once daily/52 weeks	**Inclusion:** Age>18 years, stable bronchiectasis, 3 or more LRTIs treated with oral or intravenous antibiotics, and 1 or more bacterial respiratory pathogens in the past year. **Exclusion:** >4 weeks macrolide therapy in the prior 3 months, using corticosteroid within 30 days screening or antimicrobial treatment for an LRTI in the last 2 weeks, allergy or intolerance to macrolides, childbearing without contraceptives or lactating females, liver disease or with elevated transaminase.	12 months
Serisier DJ [15]/2013	DB, RCT	117(59 treatment, 58 placebo)	Erythromycin/ethylsuccinate 400 mg twice daily/48 weeks	**Inclusion:** Age: 20 to 80 years, stable bronchiectasis, 2 or more exacerbations treated with intravenous antibiotics in the past year, and daily sputum production. **Exclusion:** CF, current mycobacterial disease or bronchopulmonary aspergillosis, any reversible cause for exacerbations, maintenance oral antibiotic prophylaxis, prior macrolide use except short-term, change to medications in the prior 4 weeks, smoking <6 months, positive sputum mycobacterial cultures, medications or comorbidities interactions with erythromycin	12 months
Masekela R [24]/2013	DB, RCT	31(17 treatment, 14 placebo)	Erythromycin/125 mg(≤15 Kg) or 250 mg(>15 Kg) daily/52 weeks	**Inclusion:** Age: 6 to 18 years, bronchiectasis associated with HIV, able to perform reliable PFTs. **Exclusion:** Abnormal liver function and urea/creatinine, use of carbamazepine, warfarin, cyclosporin or long-term midazolam, CF.	52 weeks
de Diego A [25]/2013	Open label, RCT	30(16 treatment, 14 usual care)	Azithromycin/250 mg three times a week/3 months	**Inclusion:** Adults with stable bronchiectasis. **Exclusion:** CF, pulmonary surgical processes, HIV, CVID, malignancy, emphysema, ABPA, severe liver disease or diffuse interstitial pulmonary disease, intolerance to macrolides or severe liver disease.	3 months
Valery PC [26] 2013	DB, RCT	89(45 treatment, 44 placebo)	Azithromycin/30 mg/kg once a week/12–24 months	**Inclusion:** Indigenous children with age 1–8 years, HRCT confirmed bronchiectasis or chronic suppurative lung disease, 1 or more exacerbation in the past year. **Exclusion:** long-term antibiotics, receiving chemotherapy, or immunosuppressive treatment, underlying cause for bronchiectasis (i.e. CF, primary immunodeficiency), other chronic disorders (i.e. cardiac, neurological, renal or hepatic abnormality), macrolide hypersensitivity.	12–24 months

**Abbreviation:** DB, double-blinded; RCT, randomised controlled trials; AR, airway responsiveness; CF, cystic fibrosis; ICS, inhaled corticosteroids; URTI, upper respiratory tract infection; ABPA, allergic bronchopulmonary aspergillosis; NTM, non tuberculosis mycobacteria; LRTIs, lower respiratory tract infection; PFTs, pulmonary function test; HIV, human immunodeficiency virus; CVID, common variable immunodeficiency.

The definition of bronchiectasis exacerbation differed among the included studies and was summarized in Table S1.

### Quality Assessment

Quality assessment items are shown in Table 2. Eight trials were classified as having high quality [15]–[17], [21]–[24], [26] and one as low quality [25] according to Jadad scoring system. All were randomized trials, but the methods to generate the randomization sequence were accurately reported in 5 studies [15]–[17], [23], [26]. Seven studies were double-blind and placebo-controlled trials [15]–[17], [21], [22], [24], [26] whilst the remaining studies were open-label trials [23], [25]. Seven reported concealed treatment allocation [15]–[17], [22]–[24], [26].

**Table 2:** Risk of bias assessment.

Study	Sequence generation	Allocation concealment	Blinding	Incomplete outcome data	Selective reporting	Other bias	Jadad score
Koh YY [21]/1997	unclear	unclear	low risk	low risk	low risk	low risk	3
Tsang KW [22]/1999	unclear	low risk	low risk	low risk	low risk	low risk	4
Cymbala AA [23]/2005	low risk	low risk	high risk	low risk	low risk	high risk	3
Wong C [17]/2012	low risk	low risk	low risk	low risk	low risk	low risk	5
Altenburg J [16]/2013	low risk	low risk	low risk	low risk	low risk	low risk	5
Serisier DJ [15]/2013	low risk	low risk	low risk	low risk	low risk	low risk	5
Masekela R [24]/2013	unclear	low risk	low risk	low risk	low risk	low risk	4
De Diego A [25]/2013	unclear	unclear	high risk	unclear	low risk	unclear	2
Valery PC [26]/2013	low risk	low risk	low risk	low risk	low risk	low risk	5

### The Primary outcome: the number of patients with experiencing one or more exacerbations

**Figure 2:**
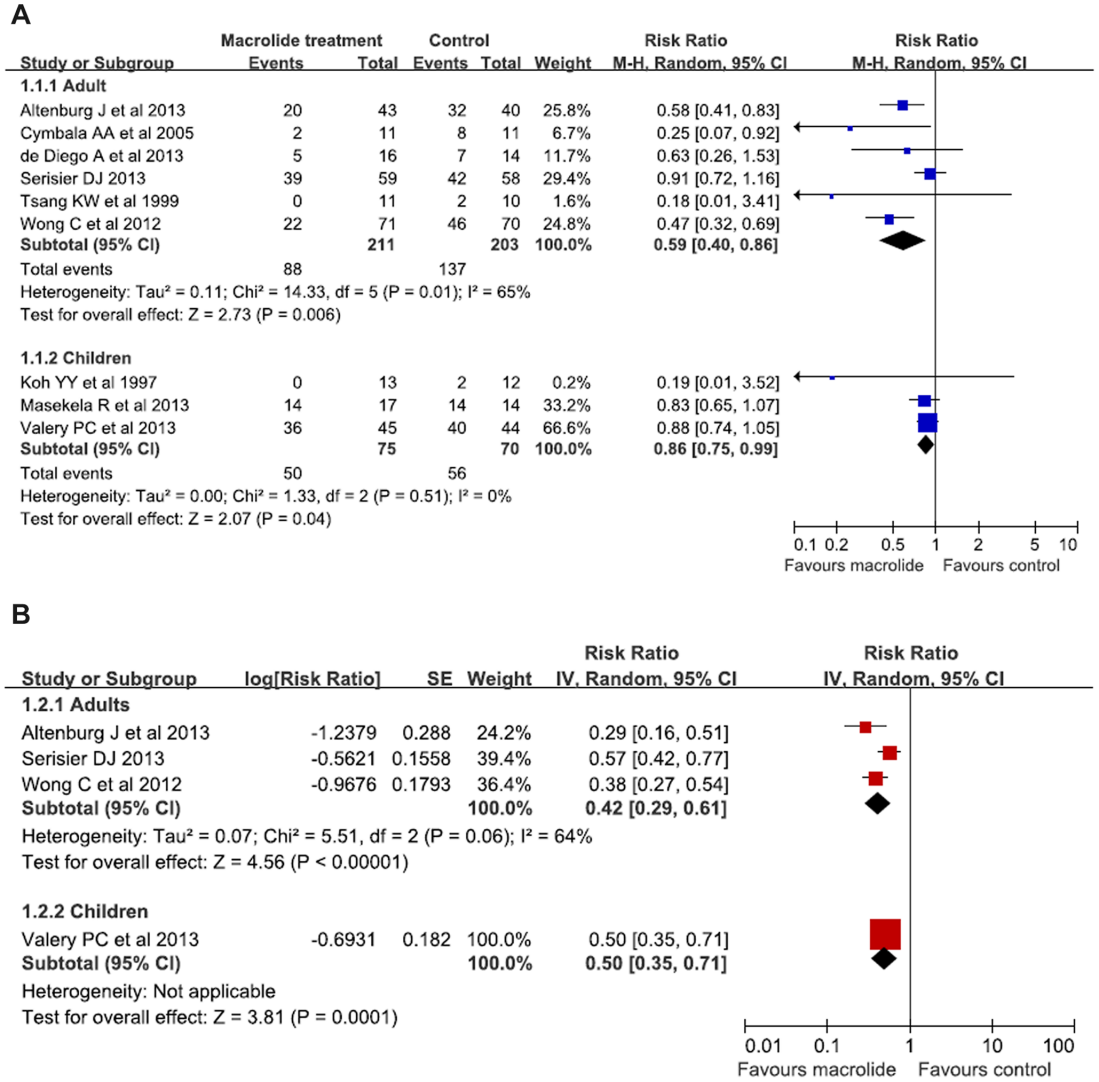
A. Meta-analysis of randomized controlled trials evaluating the effects of macrolide therapy on the number of patients with bronchiectasis exacerbations in adults and children. B. Meta-analysis of randomized controlled trials evaluating the effects of macrolide therapy on the rate of bronchiectasis exacerbation in adults and children.

The results from nine trials (n = 559) were available to examine the effects of macrolide therapy on the number of patients experiencing one or more exacerbations [15]–[17], [21]–[26]. Six trials were performed in adults and the remaining one in children. When two types of exacerbation events were reported, the one as the primary outcome was included in the pooled analysis. In adults (n = 414), pooled analyses showed that the use of macrolides was associated with a significantly reduced the number of patients experiencing one or more exacerbations (RR, 0.59; 95% CI, 0.40 to 0.86; P = 0.006; Figure 2A) with a moderate among-study heterogeneity (P = 0.01; I^2^ = 65%). Sensitivity analysis for alternative statistical model or excluding low quality studies did not markedly alter the overall findings. We further performed prespecified subgroup analyses to investigate the sources of heterogeneity (Table 3). There was a significantly greater benefit of macrolide therapy in reducing the number of patients experiencing one or more exacerbations in the following studies: longer treatment duration (RR = 0.59; 95% CI, 0.38 to 0.92; P = 0.02; I^2^ = 77%); using placebo for control (RR = 0.63; 95% CI, 0.41 to 0.96; P = 0.03; I^2^ = 74%); studies conducted in European countries (RR = 0.59; 95% CI, 0.42 to 0.82; P = 0.002; I^2^ = 0%) and United States (RR = 0.25; 95% CI, 0.07 to 0.92; P = 0.04) ; and treatment with azithromycin (RR = 0.52; 95% CI, 0.41to 0.67; P<0.001; I^2^ = 0%).

**Table 3:** Subgroup analysis of macrolide therapy on exacerbations in adults with bronchiectasis.

Variables	No. Patients	No. Trials	Macrolides	Controls	RR (95%CI)	P value	I^2^ (%)	P value for heterogeneity
**1. Number of patients with one or more exacerbation**								
**Total ** [15]–[17], [22], [23], [25]	414	6	88/211	137/203	0.59(0.40–0.86)	0.006	65	0.01
**Therapy duration**								
≥6 months [15]–[17], [23]	363	4	83/184	128/179	0.59(0.38–0.92)	0.02	77	0.004
<6 months [22], [25]	51	2	5/27	9/24	0.56(0.24–1.32)	0.19	0	0.41
**Macrolides**								
Azithromycin [16], [17], [23], [25]	276	4	49/141	93/135	0.52(0.41–0.67)	<0.001	0	0.56
Erythromycin [15], [22]	138	2	39/70	44/68	0.78(0.29–2.08)	0.61	19	0.27
**Controls**								
Placebo [15]–[17], [22]	362	4	81/184	122/178	0.63(0.41–0.96)	0.03	74	0.009
Non-placebo [23], [25]	52	2	7/27	15/25	0.45(0.19–1.08)	0.07	25	0.25
**Country location**								
United States [23]	22	1	2/11	8/11	0.25(0.07–0.92)	0.04	…	…
Asia [22]	21	1	11/21	10/21	0.18(0.01–3.41)	0.26	…	…
Europe [16], [25]	113	2	59/113	54/113	0.59(0.42–0.82)	0.002	0	0.88
Oceania [15], [17]	258	2	130/258	128/258	0.67(0.34–1.32)	0.25	89	0.003
**2. Rate of exacerbation per patients per year**								
**Total ** [15]–[17]	…	3			0.42(0.29–0.61)	P<0.0001	64%	0.06
**Macrolides**								
Azithromycin [16], [17]	…	2			0.35(0.26–0.47)	P<0.0001	0	0.43
Erythromycin [15]	…	1			0.57(0.42–0.77)	p = 0.003	…	…
**Country location**								
Europe [16]	…	1			0.29(0.16–0.51)	P<0.01	…	…
Oceania [15], [17]	…	2			0.47(0.32–0.70)	P = 0.0002	66%	0.09

In children (n = 145), macrolide therapy was also associated with decreased number of patients experiencing one or more exacerbation (RR, 0.86; 95% CI, 0.75–0.99; p = 0.04, Figure 2A), with no significant among-study heterogeneity (P = 0.51; I^2^ = 0%).

### Primary outcome: rate of exacerbations per patient per year

The exacerbation rate was expressed as the rate ratio, which was calculated using the generic inverse variance algorithm in RevMan software. Four trials involving 430 participants reported the rate of exacerbation. Three trials were conducted in adults [15]–[17] and the remaining one in children [26]. In adults (n = 341), pooled analysis showed that macrolide therapy was associated with a reduction in the rate of exacerbations (RR = 0.42; 95% CI, 0.29 to 0.61; P<0.001; Figure 2B), with a moderate level of heterogeneity (P = 0.06, I^2^ = 64%). A subgroup analysis according to the type of macrolides or the location of the country did not show any difference in the effect of macrolide therapy on exacerbation frequency (Table 3). Meanwhile, sensitivity analysis for alternative statistical model or exclusion of low quality studies did not markedly alter the overall findings.

Likewise, the use of macrolide therapy also led to reduced rate of exacerbation in children (RR = 0.50; 95% CI, 0.35 to 0.71; P = 0.0001; Figure 2B).

### Secondary Outcome: bronchiectasis exacerbation-related admissions

**Figure 3:**
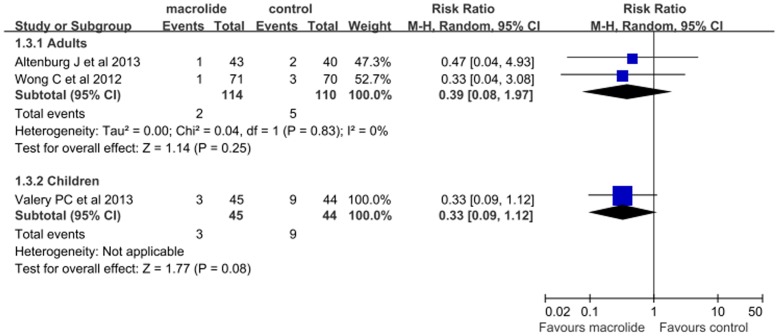
Meta-analysis of randomized controlled trials evaluating the effects of macrolide therapy on admission for bronchiectasis exacerbation in adults.

Data on exacerbation-related admissions were available in three trials (n = 313) [16], [17], [26]. Two performed in adults [16], [17], and one in children [26]. Pooled analyses in adults showed that macrolide therapy did not reduce the risk of admissions for infective exacerbations compared with control group (RR, 0.38; 95% CI, 0.08–1.94; P = 0.25; Figure 3). The heterogeneity was unremarkable (P = 0.83; I^2^ = 0%). A single study showed that exacerbation-related admission was also not significantly different between macrolide and placebo arms in children [26].

### Secondary Outcome: health-related quality of life

**Figure 4:**
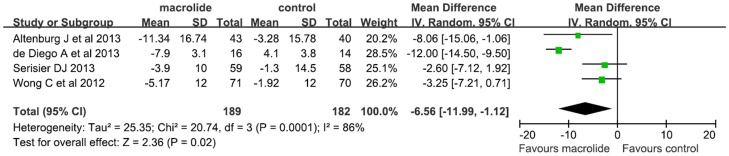
Meta-analysis of randomized controlled trials evaluating the effects of macrolide therapy on quality of life in adults with bronchiectasis.

Five trials conducted in adults (n = 393) investigated the effects of macrolide therapy on QoL [15]–[17], [23], [25]. Four assessed QoL by using St George's Respiratory Questionnaire (SGRQ) [15]–[17], [25] and the other by using subjective report [23]. Finally, four trials (n = 371) were included in the pooled analyses [15]–[17], [25]. Macrolide therapy significantly improved the SGRQ total score (WMD, −6.56 units; 95% CI, −11.99 to −1.12; P<0.001; Figure 4) compared with controls. These studies were significantly heterogeneous (I^2^ = 86%, P<0.001). Sensitivity analysis showed that removing the study with a poor quality score (Jadad score<3) [25] did not alter the results but resolved the heterogeneity (WMD, −3.75 units; 95% CI, −6.49 to −1.01; P = 0.007; P for heterogeneity  = 0.41; I^2^ = 0%).

### Secondary Outcome: spirometric indices

#### Changes in FEV_1_ from baseline

**Figure 5:**
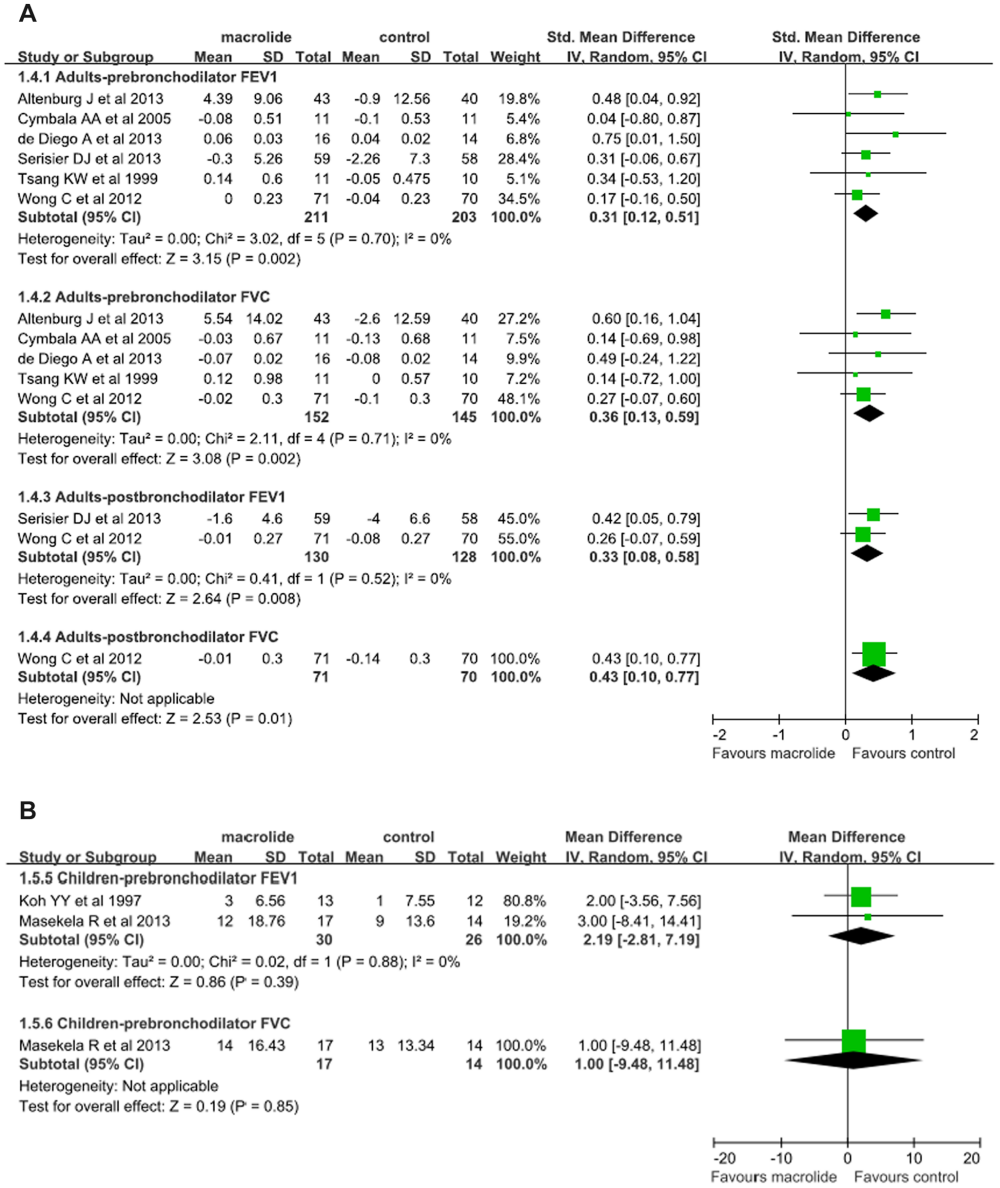
A. Meta-analysis of randomized controlled trials evaluating the effects of macrolide therapy on spirometric indices of FEV**_1_** and FVC in adults with bronchiectasis. B. Meta-analysis of randomized controlled trials evaluating the effects of macrolide therapy on spirometric indices of FEV**_1_** and FVC in children with bronchiectasis.

For Pre-bronchodilator FEV_1_, eight trials (n = 470) were available to examine the effects of macrolide therapy on pre-bronchodilator FEV_1_. Six trials (n = 414) were on adults and the remaining (n = 56) on children. In adults, pooled analyses showed that macrolide therapy yielded a significant increase in pre-bronchodilator FEV_1_ (SMD 0.31; 95% CI, 0.12 to 0.51; P = 0.002, Figure 5A) without significant among-study heterogeneity (I^2^ = 0%, P = 0.70). In children, data from two small studies showed no between-group difference (WMD 2.19; 95% CI, −2.81 to 7.19; P = 0.39; Figure 5B). Heterogeneity was not significant (I^2^ = 0%, P = 0.88).

For post-bronchodilator FEV_1_, data were only available from two studies conducted in adults showing a significant improvement between groups (SMD 0.33; 95% CI, 0.08 to 0.58; P = 0.008; Figure 5A) .without significant between study heterogeneity (I^2^ = 0%, P = 0.52).

#### Changes in FVC from baseline

For pre-bronchodilator FVC, six trials (n = 328) were included. Of which 5 were conducted on adults, and the other on children. Again, there were significant improvements in macrolide treatment groups compared with control groups (SMD 0.36; 95% CI, 0.13 to 0.59; P = 0.002; Figure 5A) without significant heterogeneity (I^2^ = 0%, P = 0.52). A single study conducted in 31 children showed no between-group differences (WMD 1.00, 95% CI, −9.48 to 11.48, P = 0.85, Figure 5B).

For post-bronchodilator FVC, data from the single study conducted in adults showed an improvement in macrolide treatment group (SMD, 0.43; 95% CI, 0.10 to 0.77; P = 0.01; Figure 5A).

### Secondary Outcome: 6-minute walk test (6MWT)

**Figure 6:**
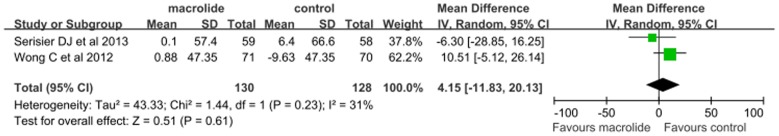
Meta-analysis of randomized controlled trials evaluating the effects of macrolide therapy on 6MWT in adults with bronchiectasis.

Only two of the nine trials (n = 258) measured 6MWT. Pooled data showed that macrolide therapy did not increase the 6MWT compared with placebo (WMD 4.15; 95% CI, −11.83 to 20.13; P = 0.61; Figure 6). The heterogeneity was not statistically significant (I^2^ = 31%, P = 0.23).

### Secondary Outcome: 24-hour sputum volume

Four trials [15], [22], [23], [25] measured the effects of macrolide therapy on sputum volume presenting with various datasets including: weight or volume, mean or median etc, rendering it difficult for pooled analyses. All trials were performed in adults. There was a significant decrease in the weight of sputum with erythromycin therapy compared with placebo in the study by Serisier et al [33] [median −4.3 g; interquartile range (IQR), −7.8 to −1]. A significant improvement in mean 24-hour sputum volume was reported with erythromycin therapy by Tsang et al [22] (33.7 ml for pre-treatment vs. 23.8 for post-treatment with erythromycin, *P*<0.05; 26.2 ml for pre-treatment vs. 22.7 for post-treatment with placebo). Cymbala et al [23] reported that the mean 24-hour sputum volume was significantly decreased [15% (P = 0.005)] during the active treatment phase, and the effects of which even persisted in control phase (P = 0.028). In one study measuring the 24-hour sputum volume [25], azithromycin was associated with reduced sputum production compared with control [−8.9 (1.8) ml with azithromycin vs. 2.1 (3.4) ml with control, P<0.05)].

### Adverse effects

Six trials (n = 473) presented data regarding adverse events [15]–[17], [22], [23], [26]. Five trials (n = 384) were on adults and the other on children (n = 89). Available data in adults are summarized in Table 4. These studies reported comparable overall adverse events (RR, 0.96; 95% CI, 0.82 to 1.13; P = 0.66; I^2^ = 0%). However, patients receiving macrolides reported significantly more events of diarrhea and abdominal discomforts, but not headache, nausea, rash or sinusitis than control group. Meanwhile, the most recent three large trials also reported other various adverse events that could not be pooled for analysis. Wong et al [17] reported similar proportions of common cold, cough and chest pain in each arm. Serisier et al [15] reported a single case of suspected corrected QT interval (QTc) prolongation in erythromycin group. Altenburg et al [16] reported similar proportions of auditory complaints, itching and palpitation in each arm. Three of the studies reported very small and similar numbers of adverse event in each arm to discontinue the trial medication. In children, the intervention drugs were well tolerated without serious adverse events.

**Table 4:** Adverse events in adults with macrolides vs. control, with summary estimates across all data.

Variables	No. Patients	No. Trials	Macrolides	Controls	RR (95%CI)	P value	I^2^ (%)	P value for heterogeneity
Any adverse events	384	5	97/195	98/189	0.96(0.83, 1.13)	0.66	0	0.58
Abdominal discomfort	224	2	13/114	2/110	6.20(1.43, 26.83)	0.01	0	0.78
Diarrhea	246	3	24/125	5/121	4.33(1.77, 10.58)	0.001	0	0.7
Headache	224	2	3/114	5/110	0.62(0.17, 2.29)	0.47	0	0.33
Nausea	341	3	15/173	14/168	1.03(0.52, 2.03)	0.93	30	0.24
Rash	94	2	9/54	5/50	1.67(0.60, 4.64)	0.33	0	0.62
Sinusitis	258	2	4/130	4/128	0.98(0.25, 3.86)	0.98	0	0.46

### Publication bias

**Figure 7:**
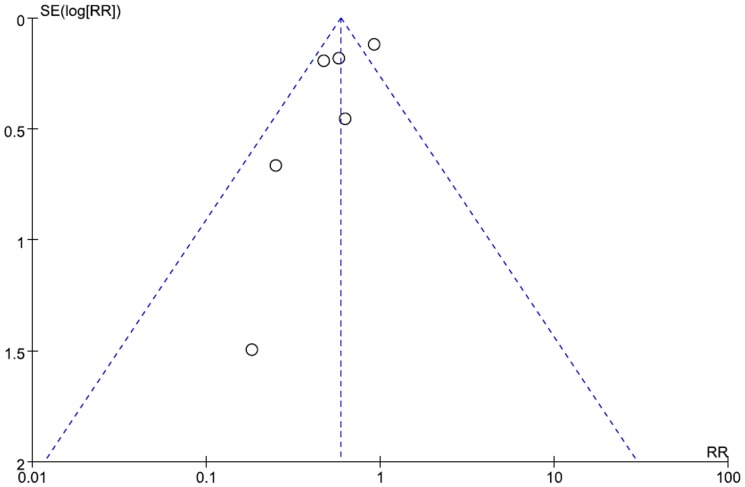
Funnel plot of included trials for bronchiectasis exacerbations in adults.

The funnel plot for the number of patients with bronchiectasis exacerbations in adults indicated a slight asymmetry (Figure 7). However, Egger's test did not show a significant publication bias (P = 0.158). Publication bias for the rate of exacerbation in adults and the exacerbations in children was not assessed due to the limited number of studies.

## Discussion

This systematic review and meta-analysis of 9 RCTs with macrolide therapy, with the duration of 2 months or more in adults and children who had stead-state bronchiectasis, indicated several meaningful findings. First, macrolide therapy significantly decreased the number of patients with exacerbations and the frequency of exacerbations, but not admissions for infective exacerbations, both in adults and children. Second, a few studies conducted in adults evidenced that macrolide therapy led to statistically significant improvement in QoL, but not 6MWT. Third, increases in FEV1 and FVC from baseline were significantly larger with macrolide therapy than with placebo or control in adults but not in children. Finally, macrolide therapy in adults might increase the risk of diarrhea and abdominal discomfort, but not overall adverse events.

We demonstrated a significantly decreased number of patients experiencing one or more exacerbations and the frequency of exacerbation with macrolide therapy in adults and children with bronchiectasis, which seemed to contradict with the 2007 Cochrane review [18]. Four recently published randomized controlled trials conducted in adults and children unanimously confirmed the beneficial effects of macrolide therapy on exacerbations. These studies contributed the majority of the weight to the pooled estimates. The subgroup analysis in adults, although not statistically significant (P = 0.44), suggested that azithromycin might have better effects on the number of patients with exacerbations than erythromycin in adults. Indeed, a recent meta-analysis [13] has demonstrated that erythromycin was associated with reduced risk of exacerbations in COPD. In addition, erythromycin was also associated with reduced rates of exacerbation. Therefore, the limited sample size, longer treatment duration and the eligible patients with a history of frequent exacerbations might help explain the aforementioned outcomes. Nonetheless, further studies are still needed to explore the true effects of erythromycin on the number of patients with exacerbations in bronchiectasis. Interestingly, the results indicated that significant benefits in reducing the number of patients with exacerbations took 6 months or more to occur in adults with bronchiectasis, which was consistent with the findings in COPD [13]. Currently, guidelines for treatment of diffuse panbronchiolitis [27] recommended a maintenance macrolide therapy for 6 months or more, and in serious conditions, a prolonged treatment. Therefore, the optimal duration of macrolide therapy should be determined in future clinical trials. While there was a significant reduction in the number of patients with exacerbations and the rate of exacerbations, macrolide administration did not reduce the exacerbation-related admissions. However, the low rate of exacerbation-related admissions reported in two trials in adults and one in children highlighted the significance of appropriately selecting clinically relevant outcomes in future trials.

The underlying mechanisms of reduced exacerbations by macrolide therapy might be in part explained by the anti-inflammatory and immunomodulatory effects that attenuate chronic airway inflammation, inasmuch that marked airway inflammation leads to a greater risk of exacerbations [28], [29]. An alternative explanation could be that macrolides alter the subtypes of P. *aeruginosa* (PA) and inhibit their adherence to respiratory epithelium as well as biofilms formation. A positive sputum culture of PA has consistently been associated with increased likelihood of infective exacerbations [29]. Macrolide therapy might have a better effect on exacerbations in patients with sputum isolation or colonization of PA than those without [15], [25]. Therefore, further studies are needed to investigate the effects of macrolides on pulmonary exacerbation by stratification of PA isolation (colonization).

Changes in health related QoL and spirometric indices also appeared statistically significant in adults with bronchiectasis. Compared with placebo or control, the mean changes in the SGRQ total score among all participants outweighed the clinically significant change of 4 units [30]. Meanwhile, pooled analyses of currently available studies showed that macrolide administration also led to improvements both in FEV_1_ and FVC. The beneficial effect for the QoL and spirometric indices might be explained at least in part by the decreased exacerbations in adults treated with macrolide. Previous studies showed that recurrent exacerbations not only led to progression deterioration of lung functions [31] but also one of the strong predictors of poor QoL [32]. The lack of beneficial effects of macrolide therapy for lung function in children might be related to limited studies (2 trials, 56 participants). Further research is warranted to investigate the definitive effects of macrolide therapy on spirometric indices in children with bronchiectasis.

Macrolide therapy did not significantly improve the 6MWT compared with placebo although the significant effects on spirometry were noted. Exercise capacity reflects not only respiratory but also systemic well-beings. Of note, exercise limitation might be not the crux in bronchiectasis, as evidenced by the median or mean for 500 meters or more reported in two studies [15], [17].

A number of studies found significant decrease in key clinical outcomes of sputum expectoration following treatment with macrolides compared to placebo or control. One potential confounding factors is the differences in the methods of measurement, with three studies using wet volume [22], [23], [25] and one dry weight [15]. The optimum approach for measuring sputum volume has not been established. Despite these limitations, these studies provided preliminary supports of macrolides in reducing sputum expectoration—a crucial symptom associated with bronchiectasis.

In terms of safety, we showed that macrolides increased the risk of diarrhea and abdominal discomforts, but not overall adverse events in adults. In practice, a major concern with using macrolides for long-term therapy in respiratory diseases is the emergence of new pulmonary pathogens and increased antimicrobial resistance of airway microbiota which should be closely monitored both in individuals and the community [33]. Macrolides for maintenance therapy in adults has not resulted in emerging pathogens but has led to increased microbial resistance [15]–[17]. Studies have suggested a significant shift in the azithromycin-treated group to carriage of macrolide-resistant bacteria in children [26]. Identification of the subgroup patients who benefits more from prolonged macrolide therapy is necessary. In addition, there were no reported serious cardiovascular events in patients treated with macrolides, which resulted in significant controversies and should be closely monitored in patients with high risk of cardiovascular disorders [34], [35]. Therefore, a balance between the improvement in crucial clinical endpoints, i.e. exacerbations, spirometry and QoL, and the development of antimicrobial resistance and adverse events should be well maintained.

Several limitations of this meta-analysis should be considered. First, there was significant among-study heterogeneity in: (1) subjects with different exacerbation history, age and disease severity; (2) the type, duration and dosage of macrolide treatment (Table 1); and (3) the definitions for exacerbation (Table S1). In fact, absence of a validated definition has precluded bronchiectasis exacerbation-related studies. However, it should be noted that all the included studies were RCTs comparing macrolide therapy with controls and the characteristics of patients were comparable. In addition, the random-effect model and subgroup analysis were used to account for this heterogeneity. Second, due to the limited number of studies and population, results from subgroups should be interpreted meticulously. Third, the results regarding the number of patients with one or more exacerbations in adults might be influenced by the publication bias. Although Egger's test did not show significant publication bias, the asymmetric funnel plot suggested that potential publication bias could not be ruled out. Recently published large RCTs unanimously demonstrated beneficial effects of macrolide therapy on reducing the exacerbations, rendering our conclusion unlikely to be altered by unpublished data. Nonetheless, more studies are needed to further confirm the findings. Finally, the largest study conducted in children with bronchiectasis included those who had not undergone CT scanning to confirm the presence of bronchiectasis.

In conclusion, macrolides had a benefit of preventing from exacerbations, but not admissions for exacerbation, both in adults and children with bronchiectasis compared with controls. Moreover, adults on macrolide therapy were associated with improvements in several other outcomes, including QoL, spirometric indices and reduced 24-hour sputum volume but not 6MWT and risk of overall adverse effects. Further studies are necessary to delineate the optimal agents, dose, duration of macrolide therapy, optimal population and potential antimicrobial resistance and cardiovascular risk.

## Supporting Information

Table S1 **The definition used of bronchiectasis exacerbation included in the meta-analysis.**
(DOCX)

Checklist S1 **PRISMA Checklist.**
(DOC)
