# Supplementary material for: Macrolide Therapy in Adults and Children with Non-Cystic Fibrosis Bronchiectasis: A Systematic Review and Meta-Analysis
Source: PLoS One. 2014 Mar 6;9(3):e90047. doi: 10.1371/journal.pone.0090047 (PMC3946068; doi:10.1371/journal.pone.0090047)
Supplement: Table S1 — The definition used of bronchiectasis exacerbation included in the meta-analysis. (DOCX) [file pone.0090047.s001.docx]

Table s1 Definition used of bronchiectasis exacerbations in the meta-analyses.

| Study/year | | Definitions |
| --- | --- | --- |
| Koh YY et al 1997 | | The following symptoms were present: Fever, increased cough and sputum production |
| Tsang KW et al 1999 | | Subjective and persistent (≥ 24 h) deterioration in at least three respiratory symptoms including cough, dyspnea, hemoptysis, increased sputum purulence or volume, and chest pain; with or without fever (≥ 37.5 ℃), radiographic deterioration, systemic disturbances, or deterioration in physical signs in the chest including crackles and dullness on auscultation and percussion, respectively. |
| Cymbala AA et al 2005 | | At least two of the following conditions: increased sputum purulence, increased dyspnea, increased volume of sputum |
| Wong C et al 2012 | | Event-based exacerbation: an increase in or new onset of more than one pulmonary symptom (sputum volume, sputum purulence, or dyspnea) requiring treatment with antibiotics.  Symptom-based exacerbation: an increase in or new onset of more than one pulmonary symptom scores (shortness of breath, sputum color, and sputum volume) from the daily dairy card on 2 consecutive days had to increase by at least one point (on a five-point scale) compared with the same calculation 1 week earlier. |
| Altenburg J et al 2013 | | Protocol-defined exacerbation (PDE): at least 4 of the following 9 symptoms, signs, or findings were present. (1) Change in sputum production (consistency, color, volume, or hemoptysis); (2) increased dyspnea (chest congestion or shortness of breath) ; (3) increased cough; (4) fever (>38℃); (5) increased wheezing; (6) decreased exercise tolerance, malaise, fatigue, or lethargy; (7) forced expiratory volume in the first second of expiration (FEV1) or forced vital capacity (FVC) decreased by at least 10% from a previously recorded value; (8) radiographic changes indicative or a new pulmonary infectious process; (9) changes in chest sounds  Non-protocol defined exacerbation: fewer than 4 of the above abnormalities. |
| Serisier DJ et al 2013 | | Protocol-defined pulmonary exacerbations (PDPE’s): 1 major and 2 minor criteria or 2 major and 1 minor criteria   1. Major criteria: increase in sputum volume or sputum purulence. 2. Minor criteria: cough, dyspnea, chest pain or haemoptysis.   Non-protocol-defined PEs: deteriorations in the respiratory symptoms that did not meet criteria for PDPEs. |
| Masekela R et al 2013 | | At least two of the following: increased tachypnea or dyspnea, change in frequency of cough, increased in sputum productivity, fever, chest pain, and new infiltrates on the chest X-ray. |
| de Diego A et al 2013 | | Clinical changes which needed antibiotics or hospital admission. |
| Valery PC et al 2013 | Treatment by clinic or hospital staff with antibiotics for any of the following: increased cough, dyspnea, increased sputum volume or color intensity, new chest examination or radiographic findings, deterioration in predicted forced expiratory volume in 1-second (FEV1) percentage by more than 10% or haemoptysis. |  |
